# The Inner Lives of Cephalopods

**DOI:** 10.1093/icb/icad122

**Published:** 2023-09-26

**Authors:** Alexandra K Schnell, Nathaniel R Farndale Wright, Nicola S Clayton

**Affiliations:** Department of Psychology, University of Cambridge, Cambridge CB2 3EB, UK; Department of Psychology, University of Cambridge, Cambridge CB2 3EB, UK; Department of Psychology, University of Cambridge, Cambridge CB2 3EB, UK

## Abstract

The minds of cephalopods have captivated scientists for millennia, yet the extent that we can understand their subjective experiences remains contested. In this article, we consider the sum of our scientific progress towards understanding the inner lives of cephalopods. Here, we outline the behavioral responses to specific experimental paradigms that are helping us to reveal their subjective experiences. We consider evidence from three broad research categories, which help to illuminate whether soft-bodied cephalopods (octopus, cuttlefish, and squid) have an awareness of self, awareness of others, and an awareness of time. Where there are current gaps in the literature, we outline cephalopod behaviors that warrant experimental investigation. We argue that investigations, especially framed through the lens of comparative psychology, have the potential to extend our understanding of the inner lives of this extraordinary class of animals.

## Introduction

Cephalopods have captivated the minds of scientists for thousands of years. Over 2000 years ago, Aristotle recorded the instantaneous color-changing abilities of cephalopods in the first modern zoological bibliography of natural history (*Historia Animalium*, 400 BC). Almost two millennia later, Charles Darwin described the skin of octopus and cuttlefish with childlike fascination in his field journal during his renowned expedition around the world (*Voyage of the Beagle*, 1839). Today, we are still enthralled by cephalopod skin, but it is their minds and inner lives that are increasingly garnering attention from the scientific community (Mather 2008, 2022; Godfrey-Smith 2017; Mather and Dickel 2017; Amodio et al. 2019; Birch et al. 2021; Crook 2021; Schnell and Clayton 2021; Schnell et al. 2021a ).

The idea that animals have inner lives with their own perspectives is not a recent concept. Almost a century ago, the German biologist Jakob von Uexküll published works that asked the reader to imagine a meadow full of different animals—butterflies, field mice, and earthworms—and envision a soap bubble around each creature to represent its private world (von Uexküll 1934). The private world draws attention to the animal’s point of view; he refers to it as their *umwelt* or self-centered, subjective world as it is sometimes translated. von Uxeküll asks us to consider the tick’s *umwelt*. It appears incredibly destitute compared to our own *umwelt*. Yet, von Uexküll argues that its simplicity is a strength: the tick’s goal is explicit and she encounters few hindrances. Half a century later, the philosopher Thomas Nagel asks, “What’s it like to be a bat.” Nagel argues that imagining the inner experience of a bat is complicated, if not impossible, especially when your reference point is the human body equipped with a human mind (Nagel 1974). In the same vein, imagining the inner lives of cephalopods seems like an unfeasible task given how radically different they are from us. Cephalopods are soft-bodied, with no skeleton, a beak for a mouth, three hearts, eight flexible arms, and a donut-shaped brain that wraps around their esophagus. How does one step into the subjective life of an octopus and apply our imagination to theirs?

Today, there is still some unease among scientists when discussing the inner experiences of animals. The apprehension does not stem from the idea that animals have inner lives—this is commonly accepted by scientists (Duncan 2006). Rather, the methods of investigating the inner lives of animals have created debate among researchers, particularly about whether the behavioral reactions of animals in response to various cognitive tasks reveal anything about subjective experience (Heyes 1994, 1995; Griffiths et al. 1999; Suddendorf and Busby 2003; Anderson and Gallup 2015). At present, there are no universally accepted behavioral markers of consciousness or subjective experience in non-human animals. As such, current evidence does not settle these debates. In the absence of behavioral markers of consciousness, researchers rely on specific paradigms that offer a window into the minds of non-verbal animals (Griffiths et al. 1999). These paradigms can be broadly classified into three categories that investigate different types of awareness: (1) awareness of self; (2) awareness of others; and (3) awareness of time. Here, we will discuss research on cephalopods within these three different categories. Where research is lacking, we discuss intriguing behaviors that deserve experimental attention since they have the potential to extend our understanding of the inner lives of cephalopods.

## Awareness of self

Self-awareness is a complex ability that is the cornerstone of human intelligence. It is defined as an understanding or recognition of self. Outside of humans, examples of self-awareness are rare. Traditionally, the mirror mark test, which investigates the ability to recognize a reflected mirror image as “self,” was considered a hallmark test of self-awareness (Gallup 1970). The test involves placing a mark on an animal in a location that can only be seen in a mirror reflection. Passing the mirror mark test involves performing self-directed behavior in the mirror, showing interest in the mark on the body, and ultimately attempting to remove the mark. In Gallup’s view, the psychologist who established the mirror mark test, there are only three species that compellingly pass the test: chimpanzees, orangutans, and humans (Anderson and Gallup 2015. However, a range of other animals, including dolphins (Reiss and Marino 2001), elephants (Plotnik et al. 2006), magpies (Prior et al. 2008), and even cleaner wrasse (Kohda et al. 2019, 2022), show self-exploratory behaviors when presented with a mirror and successfully remove visible marks. Note that the interpretation of results from mirror mark tests is hotly debated, particularly about whether the behaviors in response to the mirror can be used as evidence of self-awareness (discussed elsewhere: Heyes 1994, 1995; Anderson and Gallup 2015).

Evidence for mirror self-recognition in cephalopods is weak. Several species of octopus (*Octopus laqueus, Hapalochlaena lunulata*, and *Abdopus aculeatus*) show no response when presented with a mirror (Ikeda and Matsumura 2008). Other cephalopod species [common octopus (*Octopus vulgaris*), common cuttlefish (*Sepia officinalis*), and reef squid (*Sepioteuthis lessoniana*)] display interest toward their reflection but respond with agonistic behaviors, in a similar manner that they would respond to a conspecific (Ikeda and Matsumoto 2007; Ikeda and Matsumura 2008; Ikeda 2009; Amodio and Fiorito 2022). Notably, reef squid with visible marks tend to interact more with the mirror than sham-marked squid, but there have been no reports of squid attempting to remove the mark (Ikeda 2009). When common octopuses were marked, they explored the mark in the mirror using their arms. However, mark-directed behaviors were also observed in the absence of the mirror and in individuals that were treated with invisible sham marks. This suggests that the mark itself left an olfactory or tactile cue that evoked exploration, the presence of which is not accounted for in visually driven mark tests for self-recognition (Amodio and Fiorito 2022).

Failure to pass the mirror test does not necessarily confirm the absence of self-recognition. It has been argued that this paradigm is an inappropriate task for many animals, especially if the species in question lacks the social motivation to examine their own physical appearance, and if they are unlikely to encounter an object in their environment that has the properties of a mirror (Hauser et al. 2001). Both of these limiting aspects are relevant to cephalopods. Cephalopods are primarily solitary with limited opportunities to interact with one another in a social manner (Schnell and Clayton 2019). Consequently, inspecting a mirror reflection lacks any behavioral predisposition and might be perceived as threatening. The task also lacks ecological predispositions since cephalopods are immersed in marine environments and thus unlikely to encounter naturally occurring reflective surfaces (i.e., puddles, lakes, and rivers). Moreover, this visually centered task fails to capture a vital aspect of their sensory abilities since social recognition among cephalopods is also guided by chemical cues (Boal 2006).

Does failure to recognize oneself in a mirror suggest that all forms of self-awareness are absent in a species? Most researchers think that self-awareness is more likely to exist as a continuum in the animal kingdom with varying levels of complexity (Reiss and Morrison 2017; Lage et al. 2022). The ability to recognize oneself has an evolutionary history, unlikely to have emerged in isolation when humans evolved. Indeed, it has been suggested that different variants of self-awareness exist in the animal kingdom (DeGrazia 2009; Dale and Plotnik 2017). The most primitive form of self-awareness is bodily self-awareness (henceforth body awareness)—defined as an awareness of body parts, their position in space, and their movement. There are several paradigms that have been used to investigate body awareness in a variety of different taxa (Table 1). These paradigms were originally designed to investigate body awareness in human adults and children (Brownell et al. 2007; Costantini and Haggard 2007; Moore et al. 2007; Slater et al. 2009) and have now been adapted to test diverse animals, including elephants (*Elephas maximus*; Dale and Plotnik 2017), mice (Wada et al. 2016), snakes (*Elaphe radiata*; Khvatov et al. 2019), dogs (*Canis familiaris*; Lenkei et al. 2020), and budgerigars (*Melopsittacus undulatus*; Schiffner et al. 2014).

**Table 1:** Selection of paradigms used to investigate body awareness

Paradigm	Description
Body as an obstacle	Requires an individual to recognize their body as an obstacle to succeed in a problem-solving task.
Body size representation	Requires an individual to select appropriately sized passages to walk or crawl through, relative to their own body size.
Body ownership	Presents an individual with an illusion such as a rubber appendage (i.e., hand, tail, and arm) to induce a sense of ownership over the appendage and evoke behaviors such as stroking or grasping.

Body awareness in cephalopods has not been explicitly examined. However, cephalopods are particularly suitable for investigating body awareness because they lack an internal skeleton and an outer shell. The lack of a rigid structure allows them to adapt the shape of their body to their environment. They can squeeze into small spaces, change the length of their arms, bend their arms in all directions, and use them to manipulate objects with exceptional dexterity (Gutfreund et al. 1996; Kier 2016; Kennedy et al. 2020). The ability to fluidly change posture and overall body size suggests that cephalopods can recognize, and act upon, the distinction between oneself and their physical environment. Nevertheless, the cephalopod nervous system and body morphology, particularly those of octopuses, are radically different from those of other animals, making it challenging to investigate body awareness using paradigms designed for vertebrates. For instance, in the octopus, only the head maintains a persistent placement in space (Levy and Hochner 2017), while the arms, which possess a virtually limitless freedom of movement, can interact with the surrounding environment to support head orientation. Yet, it is thought that there is no need for central processing of complex sensory information to manipulate the location of the head (i.e., information does not travel back to the brain) (Levy and Hochner 2017). Moreover, octopus arms process and integrate tactile, chemical, and proprioceptive information. During some problem-solving tasks, information collected by the arms is fed back to the brain and combined with visual information to execute goal-directed movements (Gutnick et al. 2011, 2020). Whereas, on occasion, sensory information can be processed at the local level (Gutfreund et al. 2006) and does not reach the brain. Note that octopuses presented with different problem-solving tasks respond with exceptional flexibility and quickly adapt their motor patterns to a changing problem. Further, their solutions do not appear to rely on single fixed strategies (i.e., trial and error or stimulus–response association) (Richter et al. 2016). Such flexibility in octopus movement patterns suggests a positive correlation between motor plasticity and cognition that requires body awareness. Nevertheless, two factors need to be considered when designing whole-body paradigms to investigate body awareness: (1) octopuses are likely aware of their arms as a single entity (Levy et al. 2015); but (2) some aspects of arm coordination are not controlled by the brain and thus does not involve cognition.

**Fig. 1:**
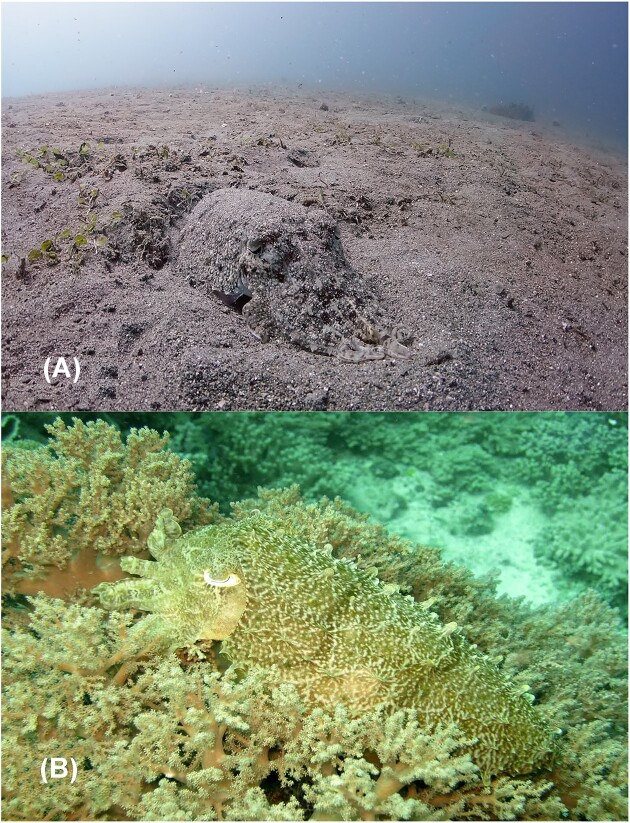
Examples of camouflage behavior in cuttlefish: (**A**) a cuttlefish camouflaged against a sandy substrate (photo: Jayvee Fernandez/Flickr/15354805307); and (**B**) a cuttlefish camouflaged with papillae extended to display a bumpy texture (photo: Lakshmi Sawitri/Flickr/10020603595).

Another potential avenue to study body awareness is through investigations of the skin. Cephalopods can dynamically change their appearance to match their surroundings. They can camouflage to nearly any visual background in their natural ranges by changing both the color and the texture of their skin (Fig. 1). Research on skin patterning in cuttlefish (*Sepia officinalis*) implies that a representation of body size is integrated into their camouflage behaviors. For example, aspects of skin patterning are determined by the size of visual cues in the environment relative to the size of their entire body (Chiao and Hanlon 2001; Josef et al. 2017). During camouflage, cuttlefish also orientate their arms to align with specific angles, which is thought to mimic aquatic plants. Arm orientation has been linked to the angle (i.e., 0°, 45°, or 90°) at which visual cues in their environment are perceived (Barbosa et al. 2012), suggesting that awareness of their own body gradient and position with respect to the surrounding environment are key elements of cuttlefish camouflage behavior.

## Awareness of others

Awareness of others is defined as the ability to recognize that others have mental states different from our own with different feelings, desires, and thoughts. This ability, commonly referred to as perspective-taking or having a theory of mind, allows an individual to take on the perspective of others in both cooperative and competitive interactions. Currently, there is no experimental evidence to suggest that cephalopods are capable of perspective-taking. Here, we instead outline intriguing behaviors observed in the wild that could potentially be driven by recognizing the mental states of others. Nevertheless, note that these behaviors require fine-grained analyses to pinpoint the underlying cognitive mechanisms.

Given that cephalopods live primarily solitary lives and do not form social bonds with conspecifics (Schnell and Clayton 2019), it seems unlikely that they would have evolved perspective-taking to mediate cooperative interactions. One exception might be the day octopus (*Octopus cyanea*), which forms collaborative partnerships with different species of fish during hunting excursions (Vail et al. 2013; Bayley and Rose 2020; Sampaio et al. 2021). Octopuses might benefit from understanding the functional role of their fish partners. Furthermore, optimal collaborators might not only understand the functional role of others but also the perspective and intention of their hunting partners. This type of awareness involves shared intentionality (i.e., collaborating with shared goals), whereby an individual forms a representation of another’s perspective and mental state (Duguid and Melis 2020). Notably, collaborative hunting can be achieved by simple rules of thumb that do not require perspective-taking but are instead driven by learning to synchronize behaviors through positive association. Perhaps little cognition is involved, and, instead, day octopuses learn to coordinate their behavior in response to a cue or signal emitted from reef fish? Further cognitive research is required to uncover the underlying mechanisms that are driving this unexpected collaboration.

**Fig. 2:**
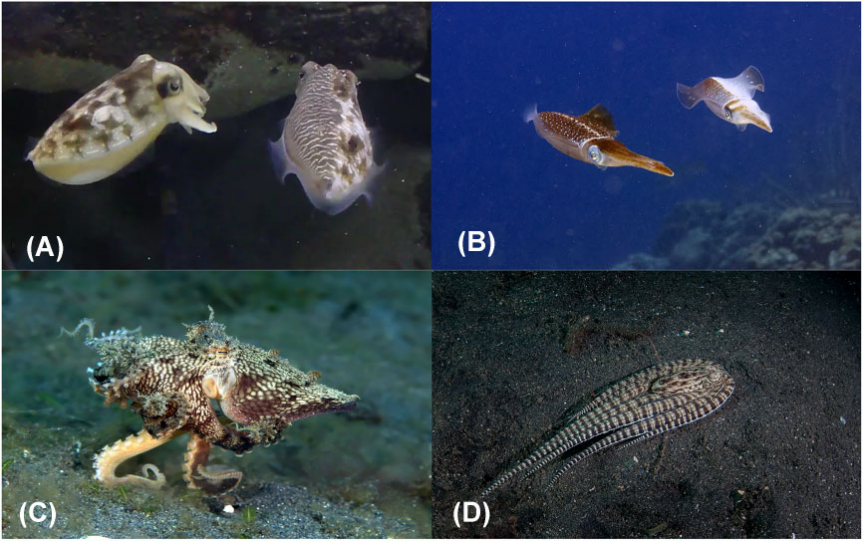
Examples of asymmetric body displays, masquerade and mimicry in cephalopods: (**A**) male mourning cuttlefish, *Sepia plangon*, (on right) produces a male-specific pattern toward a female and a deceptive female pattern toward a rival male (out of shot) (photo: Culum Brown); (**B**) male reef squid, *Sepioteuthis sepiodea*, (on right) produces a standard brown pattern toward a female and a lateral silver display to approaching male rivals (out of shot) (photo: actor212/Flickr/29454718905); (**C**) algae octopus, *A. aculeatus*, masquerades as moving algae, which is achieved by extending skin papillae to create a rough texture; and elevating all arms except for the fourth pair, which is used to walk across the substrate (photo: dynamofoto/stock.adobe.com); (**D**) mimic octopus, *Thaumoctopus mimicus*, mimics a flounder, which is achieved by flattening the body, draping all arms behind the body, and swimming low to the substrate (photo: ead72/stock.adobe.com).

Although cephalopods have limited opportunities for cooperative interactions, opportunities for competitive interactions are rife. The capacity to recognize the mental states of others would be highly beneficial in these competitive circumstances. Cephalopods appear to use deceptive resemblance to deceive conspecifics, especially during mating. Various species of cuttlefish and squid change their appearance both in color and posture to mimic members of the opposite sex (Hanlon et al. 2005; Brown et al. 2012; DeMartini et al. 2013). Some species can even change their appearance asymmetrically—across one side of their body—to deceive or communicate with a target audience (Brown et al. 2012; Hanlon and Messenger 2018; Fig. 2A and B).

Cephalopods also use deceptive resemblance to deceive potential predators. With no exoskeleton to protect them, cephalopods are highly vulnerable to predation. The dynamic ability to change their appearance allows them to use deceptive resemblances to disguise themselves as a less appealing target. When predators are nearby, some species of cephalopods can masquerade as inanimate objects such as a rock (Panetta et al. 2017) or moving algae (Fig. 2C; Huffard 2006). Other species of octopus can mimic unappetizing animals disguising themselves as marine sponges (Hanlon et al. 2008), flounder (Fig. 2D), lionfish, and banded sea snakes. Intriguingly, the mimic octopus produces different disguises in response to different target audiences (e.g., sea snake mimicry is only observed in response to an attack by territorial damselfish) (Norman et al. 2001). Both common cuttlefish and Caribbean reef squid (*Sepioteuthis sepioidea*) selectively produce defensive body patterns, which make them look larger and threatening. These defensive displays are only shown to predators likely to be intimidated or repelled (i.e., not to highly dangerous teleost fish or predators that hunt using chemical cues) (Langridge et al. 2007; Langridge 2009; Mather 2010).

Could cephalopods be taking on the perspective of others when performing these displays? Aside from cephalopods, most animals produce relatively static forms of camouflage, which are likely driven by sensory capacities and need not require cognition. In some cases, such as the horned ghost crab, *Ocypode ceratophthalmus*, camouflage is primarily controlled by endogenous rhythms and merely “fined-tuned” by sensory input with minimal opportunity for flexibility (Stephens et al. 2013). By contrast, dynamic camouflage and deceptive resemblance observed in cephalopods is performed with such flexibility that they might consider who is watching as well as the sensory capacities of their audience (e.g., how do they see sunlight, are they sensitive to polarized light). Cephalopods change color in milliseconds through color-changing skin cells called chromatophores. While several arthropods, and some fish and reptiles, also have chromatophores, only those found in cephalopods are solely under neural control (Messenger 2001). The neuromuscular nature of cephalopod chromatophores represents a practical mechanism through which their color-changing abilities may be part of wider cognitive circuits. Moreover, preliminary evidence suggests that learning (a cognitive process) might influence body patterning in cuttlefish, opening the door to a potential role for awareness in this behavior (Hough et al. 2016). The cognitive mechanisms that govern these behaviors are yet to be formally investigated. Nevertheless, these highly elaborate shape-shifting behaviors provide a unique opportunity to explore the capacity for “awareness of others” in cephalopods.

## Awareness of time

Awareness of time, also referred to as mental time travel, describes the ability to travel backward and forward in the mind’s eye to remember the past or imagine the future (Clayton et al. 2003a). In humans, the ability to recall personally experienced events from the past, or imagine scenarios in the future, is accompanied by the subjective experience of the projection of “self” in time^1^ (e.g., Tulving 1985). While we cannot currently evaluate whether this ability is associated with subjective experience in non-human animals, behavioral demonstrations of mental time travel reveal that animals share cognitive abilities with humans that are associated with awareness of time and self.

Remembering the past, also known as episodic-like memory^2^ is the former component of mental time travel that has been experimentally demonstrated in common cuttlefish. Cuttlefish can recall what they had for breakfast, where they ate it, and when they ate it (Jozet-Alves et al. 2013). Cuttlefish can also retrieve specific sensory features of episodic-like memories such as whether they had previously seen a prey item or smelt it (Billard et al. 2020a). Moreover, their episodic-like memory appears to be particularly durable. Unlike humans and other mammals, this type of memory in cuttlefish does not deteriorate with age. Adult cuttlefish nearing senescence are able to recollect temporal information to guide foraging behavior, and this continues until the final weeks of their lives (Schnell et al. 2021b).

The ability to keep track of time in cuttlefish has also shown to be exceedingly flexible. For example, cuttlefish can adjust their foraging behavior in accordance with the future availability of their preferred prey (i.e., shrimp) (Billard et al. 2020b). Specifically, cuttlefish minimize their consumption of crabs (i.e., less preferred prey) during the day when shrimp will be available at night. This flexibility relies on both previously learnt patterns of food availability and temporal expectations concerning the proximate future. Flexibility when monitoring time has also been demonstrated during foraging decisions that involve delayed gratification. Cuttlefish consider time delays when deciding whether to wait for their preferred prey or succumb to other options. Specifically, they endure certain time delays for their preferred prey. However, as delays increase, individuals begin to succumb to temptation and eat the less preferred prey that is not constrained by a time delay (Schnell et al. 2021c). Note that the maximum wait times for cuttlefish (50–130 s) are comparable to the wait times observed in apes (Beran 2002; Rosati et al. 2007), parrots (Auersperg et al. 2013), and corvids (Dufour et al. 2012; Hillemann et al. 2014). Such findings suggest that cuttlefish might monitor time delays akin to these large-brained vertebrates.

Field studies on octopuses suggest that they also use temporal information to monitor food availability. For example, octopuses avoid visiting foraging areas that they recently depleted of resources (Mather 1991; Forsythe and Hanlon 1997). However, recent research suggests that octopuses do not keep track of time like cuttlefish. They do not rely on episodic-like memory; rather, they appear to use different memory processes to encode time. Specifically, they link learned information to the order in which different events occurred (Poncet et al. 2022). In other words, the sequence of events is encoded as a proxy for time. An interesting comparison can be made with vertebrates. Jays, like cuttlefish, recall what happened, where, and when, and therefore are able to cache items that perish since they can remember when to retrieve them before they decay (Clayton and Dickinson 1998; Clayton et al. 2001, 2003b). Squirrels adopt a different strategy: they prefer to eat fresh acorns and thus avoid caching acorns that are likely to perish (Hadj-Chikh et al. 1996). Consequently, there is no need for squirrels to keep track of time in decisions about what to cache for the future. From these experiments, we can infer that jays have a greater awareness of time than squirrels when it comes to caching behavior. While octopuses appear to monitor time through different cognitive processes, further research is required to pinpoint whether cuttlefish have a greater awareness of time than octopuses. One way of investigating this question would be to explore whether cuttlefish can keep track of items that decay at different rates, akin to jays, and whether they can adjust their search behavior after a time delay based on new information that they received following their initial foraging period (see Clayton et al. 2003b).

## Conclusion

The inner lives of cephalopods are likely to be highly different from our own; thus, we might never know what it is truly like to be an octopus, cuttlefish, or squid. Nevertheless, trying to understand their world from their point of view will not only offer valuable implications for welfare—a pressing matter given current plans for commercial farming (Marshall 2023)—but might reveal important details about the evolution of our own minds. We encourage researchers to investigate cephalopod behaviors using the paradigms outlined in this article so that we can begin to create a broad cognitive catalog to understand their inner lives. The application of these psychological paradigms offers a window into the cephalopod mind, adapting non-invasive avenues to understand whether they are aware of themselves, aware of others, and aware of time.

## Data Availability

There are no new data associated with this article.

## Footnotes

1 The projection of self in time is linked to two phenomenological characteristics of consciousness that claim to be an integral part of mental time travel in humans: (1) autoneosis (self)—awareness of ownership of a memory; and (2) chronesthesia (time)—an awareness of the passage of time. Together these create an awareness of the projection of self in time.

2 In humans the capacity is termed episodic memory. In non-human animals we use the term episodic-like memory because we have only assessed this type of memory through behavioral criteria, which cannot be confirmed by verbal reports of conscious experiences.
